# On the Identification and Quantification of Ergothioneine and Lovastatin in Various Mushroom Species: Assets and Challenges of Different Analytical Approaches

**DOI:** 10.3390/molecules26071832

**Published:** 2021-03-24

**Authors:** Konstantinos Tsiantas, Thalia Tsiaka, Georgios Koutrotsios, Eleni Siapi, Georgios I. Zervakis, Nick Kalogeropoulos, Panagiotis Zoumpoulakis

**Affiliations:** 1 Institute of Chemical Biology, National Hellenic Research Foundation 48, Vas. Constantinou Ave., 11635 Athens, Greece; ktsiantas@uniwa.gr (K.T.); thtsiaka@eie.gr (T.T.); esiapi@eie.gr (E.S.); 2 Department of Food Science and Technology, University of West Attica, Ag. Spyridonos, 12243 Egaleo, Greece; 3 Laboratory of General and Agricultural Microbiology, Department of Crop Science, Agricultural University of Athens, 11855 Athens, Greece; georgioskoutrotsios@gmail.com (G.K.); zervakis@aua.gr (G.I.Z.); 4 Laboratory of Chemistry, Biochemistry, Physical Chemistry of Foods, Department of Nutrition and Dietetics, School of Health Science and Education, Harokopio University, 70 El. Venizelou Str., 17661 Athens, Greece; nickal@hua.gr

**Keywords:** mushrooms, ergothioneine, lovastatin, ultraviolet–visible spectroscopy (UV–Vis), liquid chromatography–mass spectrometry (LC–MS)

## Abstract

In recent years, mushrooms have drawn the attention of agro-industries and food-industries as they were considered to be valuable natural sources of health promoting compounds such as β-glucans, ergothioneine, and lovastatin. The detection and quantification of such compounds by implementing reliable analytical approaches is of the utmost importance in order to adjust mushrooms’ cultivation conditions and maximize the production in different species. Toward this direction, the current study focuses on the comparison of ultraviolet–visible (UV–Vis) spectrometry and liquid chromatography–mass spectrometry (LC–MS) methods (a) by evaluating the content of ergothioneine and lovastatin in mushrooms and (b) by highlighting any possible substrate-based interferences that hinder the accurate determination of these two compounds in order to propose the technique-of-choice for a standardized bioactive compounds monitoring. For this purpose, mushrooms produced by three species (i.e., *Agaricus bisporus*, *Pleurotus ostreatus*, and *P. citrinopileatus*) on various cultivation substrates, namely wheat straw (WS), winery (grape marc (GM)), and olive oil (OL) by-products, were examined. Among the two applied techniques, the developed and validated LC–MS methods, exhibiting relatively short analysis time and higher resolution, emerge as the methods-of-choice for detecting ergothioneine and lovastatin in mushrooms. On the contrary, UV–Vis methods were hindered due to co-absorbance of different constituents, resulting in invalid results. Among the studied mushrooms, *P. citrinopileatus* contained the highest amount of ergothioneine (822.1 ± 20.6 mg kg^−1^ dry sample), whereas *A. bisporus* contained the highest amounts of lovastatin (1.39 ± 0.014 mg kg^−1^ dry sample). Regarding the effect of different cultivation substrates, mushrooms produced on OL and WS contained the highest amount of ergothioneine, while mushrooms deriving from GM-based substrates contained the highest amount of lovastatin.

## 1. Introduction

From the ancient years, mushrooms have been an integral part of many different culture diets such as the Asian, the European, and the American. Besides that, mushrooms were the basic ingredients of ethno-pharmacology and folklore medicine [1], since they exert several health-promoting properties, such as antioxidant, anti-inflammatory, anti-cancer, antimicrobial, anti-cholesterol, prebiotic, geno-protective, and immunomodulating activities, which are associated with specific compounds present in mushrooms, like ergothioneine, polysaccharides (chitosan, β-glucan), terpenes, lectins, and lovastatin [2,3,4,5,6,7,8]. Although the beneficial effects of mushroom consumption are well-established, their biological activities and mechanism of action varies among species [9].

Currently, the advances on agricultural practices and the introduction of modern non-conventional approaches have improved the efficiency of mushroom cultivation. As a result, more than thirty mushroom species are commercially cultivated, and the production of more than twenty are currently on the scaling-up stage [10]. According to Food and Agriculture Organization (FAO) statistics, mushroom production shows an increasing trend of about one million tons per year, disclosing a huge socioeconomical and commercial impact at a global level [11]. Species of the genera *Agaricus* (white button mushroom) and *Pleurotus* (oyster mushroom) are among the top-five in the world mushroom supply [12].

Focusing on mushrooms’ secondary metabolites, ergothioneine and lovastatin are important metabolites of fungal growth with well-established bioactive properties. Therefore, optimization of the cultivation conditions and practices is of the utmost importance [13]. Ergothioneine (ESH) is a water-soluble thiol compound, whose composition involves the amino acids histidine, cysteine, and methionine [14]. In recent years, ergothioneine held researcher’s attention because of its beneficial effects against autoimmune disorders, such as rheumatoid arthritis and Chron’s disease, that are strongly related to ergothioneine’s antioxidant properties [15]. According to current in vitro studies, decreased blood and tissue levels of ergothioneine have been observed in some diseases, such as chronic inflammatory conditions, cardiovascular disorders, and ischemia, suggesting that ergothioneine can play a pivotal protective role in various pathological conditions [16].

Lovastatin (LOV) is a natural statin, mainly produced by *Aspergillus terreus* strains [17]. It is widely known, over years, that statins can lower total and low-density lipoprotein (LDL) cholesterol levels and reduce the risk of coronary heart disease by competitively inhibiting 3-hydroxy-3-methylglutaryl coenzyme A (HMG CoA) reductase, which is a critical rate-limiting enzyme in the production of cholesterol [18,19]. In addition to the previously mentioned main action of statins, lovastatin has revolutionized the treatment of hypercholesterolemia and it is proven to be therapeutically and preventatively effective in the treatment of major types of diseases, like atherosclerosis, sepsis, peripheral arterial and vascular disease, cerebral vascular disease, ischemic disease, and bone fracture [20]. Lovastatin in mushrooms can be present in its lactone form or in its hydroxyl metabolite. This bioconversion is bidirectional and significantly affected by the prevailing pH conditions [21]. At a low pH, most of the acidic form is converted to the lactone-quantifiable lovastatin even though the equilibrium is still present [22]. Therefore, cultivation conditions, special pre-treatments, or pH adjustments during analysis can affect the identified form of lovastatin.

The development and validation of robust, fast, accurate, and reliable analytical methodologies to determine the actual concentration of ergothioneine and lovastatin by sidestepping any possible interferences or errors generated by other co-existing mushroom constituents is of major importance. According to already published works, both ergothioneine and lovastatin absorb light at 238 and 254 nm, respectively. Until now, there have been no published studies where ultraviolet-visible (UV–Vis) spectroscopy was implemented for detecting ergothioneine and lovastatin as a stand-alone technique.

Spectrophotometric detectors, like a diode array detector (DAD), coupled with liquid chromatography (LC), have been extensively used for the identification and quantification of the two analytes in mushrooms [23]. In most cases, the applied liquid-chromatography ultraviolet (LC–UV) approaches are time consuming (analysis time >20 min), not fully validated methods, that may lead to an under-estimation or over-estimation of the relatively low (compared to other mushrooms metabolites) ergothioneine and lovastatin content [24,25]. Accordingly, the hyphenation of faster LC technologies of improved resolution ability with mass spectrometry (MS) promotes enhanced sensitivity, higher selectivity, and higher sample throughput of the LC–MS technique, compared to high-pressure liquid chromatography with ultra-violet detector (HPLC–UV) methods. Up to date, the assets of LC–MS methods are reflected particularly in ergothioneine and lovastatin analyses in dietary supplements, blood, and different human body tissues [26,27]. Nonetheless, this methodology has not been broadly applied to determine ergothioneine and lovastatin content in mushrooms where the presence of other co-extracted molecules may hinder the export of unbiased results.

One of the benchmarks of the present study was to assess the good performance of a simple, fast, low-cost, easily portable, in-the-field measurements-technique, like UV-Vis spectrophotometry, by (i) juxtaposing it with LC-MS outcomes and (ii) evaluating if the spectrophotometric approach could provide equally reliable and valid results since there are no reports that examine if UV-Vis is suitable or not for determining ergothioneine and lovastatin. Thus, two different analytical methodologies based on UV–Vis and LC–MS techniques were developed and compared in order to (a) appoint the method/technique-of-choice for the analysis of bioactive compounds present in low concentrations, such as ergothioneine and lovastatin in mushrooms of three species (i.e., *Agaricus bisporus*, *Pleurotus ostreatus*, and *P. citrinopileatus*) and reveal any possible interferences that impede their determination including the use of various cultivation substrates (in the case of *P. citrinopileatus*), and (b) compare and identify mushroom species with higher content of ergothioneine and lovastatin. Overall, results of this study contribute to the appropriate selection of mushroom species along with the methodical choice of optimal cultivation conditions, which shape the final content of mushrooms in different bioactive compounds.

## 2. Results and Discussion

### 2.1. Results of Validation of LC-MS Methods

Analytical figures of merit of LC-MS analysis for ergothioneine and lovastatin are presented in Table 1.

The results for repeatability, reproducibility, accuracy, and a matrix effect at three levels of concentration (quality control-QC samples) for both methods are summarized in Table 2. Since the present study does not address the analysis of biological samples or drugs, relative standard deviation (RSD%) values for repeatability and intermediate precision were satisfactory for both methods, not exceeding the maximum acceptable value of 15% [28]. Process recoveries for ergothioneine, regarding all mushroom species and substrates, ranged from 75.0% to 85.0%. Accordingly, extraction recovery for lovastatin varied from 63.0% to 79.2%.

### 2.2. Determination of Ergothioneine and Lovastatin Content of Mushrooms Produced in Conventional Substrates

Ergothioneine and lovastatin contents of different mushrooms, produced in conventional substrates (wheat straw and manure for *A. bisporus*, wheat straw for *Pleurotus* spp.), by using UV–Vis and LC–MS, is presented in Table 3.

According to LC–MS results, *p-*values indicated that ergothioneine contents differed significantly among all three species, offering a clear discrimination of the studied mushrooms based on their identity. In contrast, UV–Vis analysis sorted the investigated samples into two different groups based on ergothioneine concentration. Furthermore, it can be noted that ergothioneine concentration was an order of magnitude higher when UV–Vis method was applied, implying the existence of a possible positive error in spectrophotometric measurements.

Comparisons among the three mushrooms examined, which are demonstrated in both methods, *P. citrinopileatus* contained the highest amounts of ergothioneine, indicating that this particular species is more suitable for an ergothioneine-oriented mushroom production (Table 3). Several comparative studies report that *Pleurotus* species and *P. ostreatus* contain higher concentrations of ergothioneine compared to other edible mushrooms [23,29]. This is possibly associated with differences or changes in the biosynthetic pathways, which are responsible for the formation of ergothioneine, among mushroom species. Moreover, another important factor appears to be the bioavailability of compounds that can activate ergothioneine biosynthesis. For instance, the higher ergothioneine content of *P. ostreatus* may be ascribed to the higher levels of the precursor molecules histidine, cysteine, and methionine, that *P. ostreatus* contain, when compared to *A. bisporus* [30].

Similarly, based on lovastatin content measured by LC–MS, the samples were classified into three different groups, whereas, according to UV–Vis results, no statistically significant difference was observed among the mushrooms examined (*p-*values > 0.05) (Table 3). As in the case of ergothioneine’s spectrometric determination, lovastatin content using UV–Vis was over-estimated. *Agaricus bisporus* showed a higher amount of lovastatin than both *Pleurotus* species. However, *A. bisporus* and *P. ostreatus* lovastatin concentrations did not deviate much (Table 3). Nonetheless, the *P. citrinopileatus* strain used, which produced fruit bodies with a high content in ergothioneine, would not be proposed for the cultivation of lovastatin-rich mushrooms. These differentiations can be affected by many factors, such as the fungal strain and/or the substrate, since lovastatin production is crucially affected by the content of carbon and nitrogen [31], differences in gene adjustment, and bioavailability of compounds that can activate lovastatin biosynthesis, such as methionine, glutamate, glycine, and histidine [32].

### 2.3. Determination of Ergothioneine and Lovastatin Content of P. citrinopileatus Mushrooms from Different Substrates

Since *P. citrinopileatus* is a species that has not been thoroughly investigated, it was the one selected to be cultivated not only in the commonly used wheat straw (WS) substrate, but also in two “non-conventional” cultivation substrates, namely grape marc (GM) and olive by-products (OL). Ergothioneine and lovastatin content in the derived mushrooms were measured by using UV–Vis and LC–MS (Table 4).

It’s interesting to mention that UV–Vis results showed no statistically significant differences (*p-*value > 0.05) in both ergothioneine and lovastatin contents among mushrooms from three substrates. On the other hand, LC–MS methods indicated that *P. citrinopileatus* produced in OL contained the highest amount of ergothioneine (although differences were not significant versus those of mushrooms deriving from WS), while *P. citrinopileatus* cultivated in GM exhibited the highest concentration of lovastatin (Table 4). These results revealed that the nature of growth substrate can play an important role in ergothioneine and lovastatin biosynthesis of *P. citrinopileatus*. It was observed that ergothioneine and lovastatin levels detected in GM substrates in a polyphenol-rich matrix differed significantly from those determined in WS and OL (Table 4). This is likely due to suppression (ergothioneine) or overexpression (lovastatin) mechanisms involved in the pertinent biosynthetic pathways, resulting in low ergothioneine and high lovastatin content in GM-cultivated mushrooms, respectively.

However, it is possible that these mechanisms may also be associated with various bioactive compounds present in the substrates examined. These compounds can act either as precursors, inducers, or inhibitors of the examined analytes biosynthesis affecting ergothioneine and lovastatin final content in mushrooms [33]. The differences in the type and nature of phenolic compounds and amino acids contained in each one of the studied agricultural by-products may be considered as the key factor for the under-production or over-production of the investigated compounds. However, this is not underlined evidently in existing literature, and further research is required in order to shed light on the effect of substrates’ bioactive compounds in the production of ergothioneine and lovastatin. Apart from that, these additional bioactive compounds (i.e., polyphenols, amino acids, etc.) of the growth medium can be absorbed by the mushrooms, increasing not only their nutritional but also their added value [34].

### 2.4. Comparing Analytical Approaches for the Identification and Quantification of Ergothioneine and Lovastatin in Mushrooms

#### 2.4.1. Ultraviolet-visibleSpectroscopy (UV–Vis)

**Figure 1:**
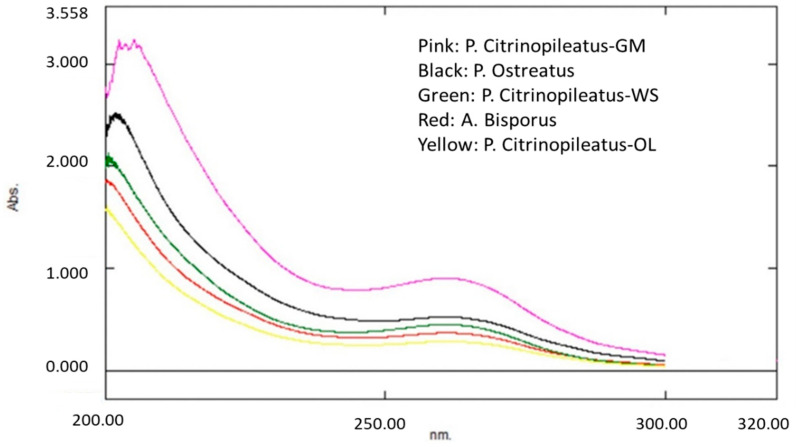
Ergothioneine ultra-violet spectroscopy (UV–Vis) spectra of mushrooms of three species, i.e., *A. bisporus*, *P. ostreatus*, and *P. citrinopileatus*. The latter was cultivated in three substrates (wheat straw (WS), grape marc (GM), and olive by-products (OL)).

As already mentioned, several bioactive compounds absorb in the wavelengths analyzed for ergothioneine and lovastatin, increasing the possibility of providing false positive errors in the final measurements. More specifically, ergothioneine was analyzed at 260 nm (Figure 1), which is a region of the spectrum in which some nucleic acids and aromatic amino acids (present in mushrooms) absorb light [35,36]. It is known that nucleic acids represent large amounts of nonprotein nitrogen in fungi [30]. In addition, *Pleurotus* mushrooms and grape marc by-products contain a higher amount of the aromatic amino acid phenylalanine, which may absorb at the same wavelength used for ergothioneine’s spectrophotometric determination, leading to an erroneously increased ergothioneine’s peak area (Table 3 and Table 4).

In contrast, *Agaricus* mushrooms and olive by-products do not contain equally high concentrations of such amino acids [37,38]. This may be related to the lower ergothioneine content measured in *A. bisporus* and *P. citrinopileatus*-OL mushrooms by UV–Vis spectroscopy (Table 3 and Table 4). In addition, phenolic content in mushrooms or in their cultivation substrates can affect UV–Vis quantification. These positive errors may affect more *P. citrinopileatus* samples rather than other mushrooms produced on WS-based substrates, since *P. citrinopileatus* samples are also derived from agricultural by-products that contain significant amounts of phenolic compounds [39,40]. Especially, wines and wineries residues, like grape marc, contain quercetin rhamnoside, kaempferol, hydroxybenzoic acid derivatives, and myricetin 3-O-glucoside that can absorb in similar wavelengths to that of ergothioneine’s UV–Vis determination, explaining the excessively higher concentrations of ergothioneine presented in Table 5 [41,42].

Regarding the levels of lovastatin detected, it should be noted that there are some intermediates of the lovastatin biosynthesis pathway and some structural analogues of lovastatin that can absorb light at 232–238 nm, i.e., the wavelengths selected for lovastatin’s UV–Vis analysis [43]. These intermediates are mostly degraded compounds, such as methyl esters, anhydro, methoxy, and acetate ester forms of lovastatin, that can absorb in those wavelengths due to the diene groups they include [44]. Similarly, olive by-products (olive leaves and olive mill waste) contain tyrosol, hydroxytyrosol, apigenin, and *p-*coumaric acid hexoside that can absorb in similar wavelengths to lovastatin [45].

**Figure 2:**
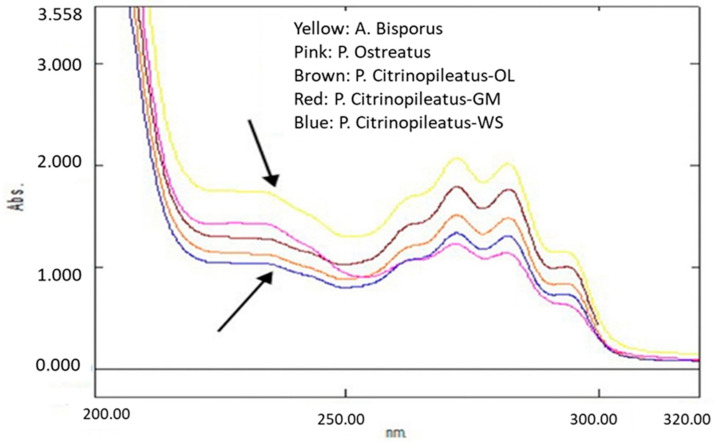
Lovastatin UV–Vis spectra of three species, i.e., *A. bisporus*, *P. ostreatus*, and *P. citrinopileatus*. The latter was cultivated in three substrates (WS, GM, and OL).

These hypotheses could be supported by the results of the present work (Table 3 and Table 4). Moreover, by comparing the UV–Vis spectra of pure lovastatin from literature [46] with the corresponding UV–Vis spectra of the mushroom samples examined (Figure 2), it was noticed that the hydroxyl metabolite of lovastatin likely coexists with the lactone form. This can be assumed by the presence of one broad peak instead of two separate sharp peaks, with one for lovastatin and one for lovastatin acid, which is a highly unstable metabolite that can easily be converted to the lactone form. As proven by the interpretation of the results of UV–Vis analysis, a more selective technique, like LC–MS, may be more suitable for the quantification of ergothioneine and lovastatin in order to avoid a possible interference.

#### 2.4.2. Liquid Chromatography–Mass Spectrometry (LC–MS)

Ergothioneine contents determined in *P. ostreatus* and *P. citrinopileatus-*WS (Table 3), by the developed LC–MS method, were similar to those reported in other studies, such as Lin et al. [47], who found a 997 mg ergothioneine kg^−1^dry sample by using a high-pressure liquid chromatography with a diode array detector (HPLC–DAD) method. Additionally, the LC–MS results of our study were in accordance with results provided by Weigand-Heller et al. [48], who also implemented an LC–MS methodology to evaluate ergothioneine’s content in *Agaricus* mushrooms.

*P. citrinopileatus* samples, which were produced in OL, demonstrated the highest ergothioneine content than any other mushroom sample, irrespectively of species or a cultivation substrate, while *P. citrinopileatus*-GM along with *A. bisporus* mushrooms contained the lowest content (Table 3 and Table 4). As already stated, this finding is possibly related to the different compounds that non-conventional growth substrates contain including phenolic compounds and amino acids. Both amino acids and polyphenols hindered the UV–Vis determination of the two analytes due to their co-absorbance at specific wavelengths.

Lovastatin contents of the studied mushrooms were considerably lower than that reported in other studies [23,24]. The present outcome seems to be related to the greater sensitivity of the Orbitrap MS instrumentation used for lovastatin determination compared to the mass detectors used in the other studies. Relying on the higher resolution and sensitivity of our validated LC–MS methodology, we can assume that it can quantify or even detect lovastatin in samples of extremely low content.

The need to apply a more sensitive and accurate technique for the evaluation of lovastatin content is also underscored by reviewing the presently published literature. Although, in the current study, lovastatin was detected in all mushroom samples, there are conflicting reports concerning the content of lovastatin in *P. ostreatus and P. citrinopileatus.* For example, Lam & Okello [25], Lin et al. [47], and Cohen et al. [49] did not detect lovastatin in *P. ostreatus* and *P. citrinopileatus* mushrooms, while Lo et al. [24] determined high concentrations of the same analyte. Along with the applied detection methodology, different cultivation practices (strain type, pH, aeration, temperature, and incubation period) as well as methods of extraction and measurement could also affect lovastatin production and determination.

To sum up, LC–MS platforms emerge as the method-of-choice for the accurate determination of both ergothioneine and lovastatin since they circumvent the interferences and drawbacks of UV–Vis protocols. Taking into consideration the results of the developed LC–MS methods, *P. citrinopileatus* could be considered an efficient alternative to the most common commercial species (i.e., *A. bisporus* and *P. ostreatus*) for the production of ergothioneine-rich mushrooms. Even though *P. citrinopileatus* would not be selected as the most appropriate species (on the basis of the outcome provided by the single strain examined) for producing mushrooms rich in lovastatin, its cultivation in novel non-conventional substrates, e.g., based on grape marc, can raise the final lovastatin content, highlighting the exploitation potential of such by-products in mushroom cultivation.

## 3. Materials and Methods

### 3.1. Reagents and Standards

Standards of lovastatin and simvastatin (SIMV, Internal Standard, IS) were purchased from European Pharmacopoeia (purity >98%, Strasbourg, France). Ergothioneine was purchased from Sigma Aldrich (purity >99%, St. Louis, MO, USA), while methimidazole (METH, IS) was purchased from Thermo Fisher (purity >99%, Erlenbachweg, Germany).

All standard stock solutions of lovastatin, simvastatin, and methimidazole were prepared in acetonitrile, while acetonitrile-water 7:3 (% *v*/*v*) was used to dissolve the water-soluble ergothioneine. The prepared stock solutions were stored at −18 °C. All solvents were of an LC–MS grade. Acetonitrile was purchased from Sigma Aldrich (St. Louis, MO, USA) and water was from Sharlau (Barcelona, Spain). Methanol was provided by ChemLab (Zadeglem, Belgium), while formic acid was obtained from Fisher Scientific (Hampton, VA, USA).

### 3.2. Biological Material–Mushroom Cultivation

Mushrooms of three species (Fungi and Basidiomycota) were examined in this study. Those of *Pleurotus ostreatus* and *Agaricus bisporus* were purchased from a local market, while *P. citrinopileatus* was cultivated at the Laboratory of General and Agricultural Microbiology, Agricultural University of Athens. Three substrates were used for this purpose, i.e., wheat straw (WS), grape marc plus wheat straw (GM; 1:1, *w*/*w*), and two-phase olive mill waste plus olive leaves (OL; 1:1, *w*/*w*). Their preparation process, the inoculation of the fungal strain (LGAM 158), and the conditions for mushroom production were previously described [50]. Grape marc was obtained by a winery located in Nemea (Peloponnese). Olive leaves and two-phase olive mill waste were obtained from an olive mill located in Kalamata (Peloponnese), and wheat-straw was kindly provided by Dirfis Mushrooms SA (Euboea).

### 3.3. Sample Preparation

After cultivation, whole mushrooms were collected and frozen to −20 °C for one day and freeze dried in a ModulyoD Freeze Dryer, equipped with a Thermo Savant ValuPump VLP200 (Thermo Electron Corporation, Thermo Fischer, Waltham, MA, USA). Freeze drying was selected as the optimum drying method since it protects sensitive metabolites and bioactive compounds from degradation during long-term storage. This method removes samples’ moisture that may produce undesirable chemical reactions and promote microbial growth [51]. Prior to analyses, dried material was homogenized and powdered in a laboratory mill (Type ZM1, Retch GmbH, Haan, Germany). Dry material and all samples and extracts were kept in airtight packaging bags and vials at −20 °C.

### 3.4. Extraction Procedure

The extraction process applied for the ergothioneine recovery was based on an already published protocol [29], slightly modified with regard to the centrifugation conditions. Ergothioneine was extracted from 100 mg of dried mushroom powder with 10 mL of 1:4 (% *v*/*v*) aqueous methanol by vigorous shaking for 20 min in a vortex (Falc Instruments, Bergamo, Italy), which is followed by centrifugation (Centrifuge Z32 HK, Hermle, Wehingen, Germany) at 3650 rcf for 20 min. After centrifugation, 8 mL of the supernatant were placed in the freeze dryer in order to acquire the dry residue of the extracts.

The lovastatin extraction procedure was based on a previously developed extraction process with slight modifications [23]. Lovastatin was extracted from 400 mg dried mushroom powder with 4 mL of acetonitrile followed by vigorous shaking (Falc Instruments, Italy). This was followed for 2 h at 250 rpm. The extract was then centrifuged for 20 min at 3650 rcf. Three mL of the supernatant were evaporated using a nitrogen pump to remove the extraction solvent.

### 3.5. Ergothioneine–Lovastatin Analysis

#### 3.5.1. Ultraviolet–Visible Spectroscopy (UV–Vis)

**Figure 3:**
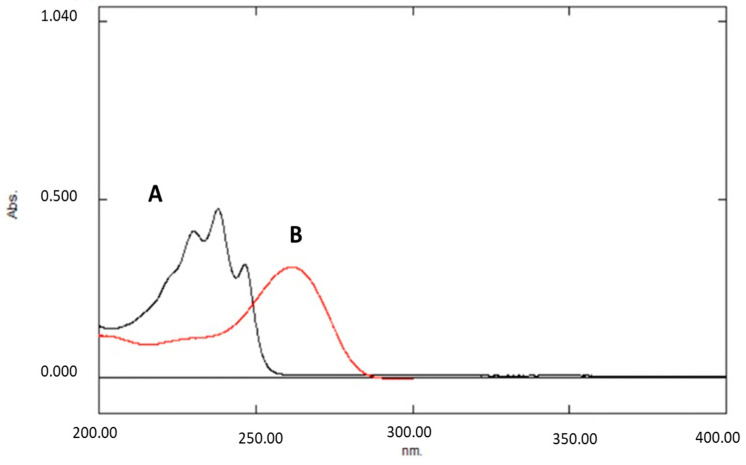
Lovastatin absorption peaks at 232, 238, and 247 nm (A) and ergothioneine absorption peak at 260 nm (B).

Ultraviolet–Visible (UV-Vis) analysis was conducted by using a dual beam spectrophotometer (UV- 1900, Shimadzu Corporation, Kyoto Japan), while scanning from 200 to 400 nm was performed to determine maximum wavelengths. After reviewing the spectra of the two investigated compounds, ergothioneine analysis was performed at 260 nm, while lovastatin analysis took place at 232 nm instead of 238 nm in order to minimize possible interferences (Figure 3).

The linearity of calibration curves was determined by using standard solutions of the two compounds with concentrations ranging from 1 to 20 μg mL^−1^ for both ergothioneine and lovastatin. Coefficient factors (R^2^) were 0.9979 for ergothioneine and 0.9988 for lovastatin, verifying the method’s linearity. Two to five (2–5) milligrams of each mushroom extract’s dry residue were dissolved in 10 mL of 3:7 (*v*/*v*) methanol–water for ergothioneine and in 5 mL of acetonitrile for lovastatin. All spectra were processed by UV Probe software (2.7 version, Shimadzu Corporation, Kyoto, Japan).

#### 3.5.2. Liquid Chromatography–Mass Spectrometry (LC–MS)

Liquid chromatography mass spectrometry (LC–MS) analysis was used for the identification and quantification of ergothioneine and lovastatin in mushroom species. The instrumentation of liquid chromatography for both methods included a quaternary pump, an autosampler with a tray oven set at 25 °C (Accela, Thermo Scientific, Waltham, USA), and a guard column. For ergothioneine analysis, a Kromacil C18 column (3.5 μm particle size, 100 × 2.1 mm i.d.) was used at 25 °C, while lovastatin separation was performed by an Acquity C18 column (1.7 particle size, 100 × 2.1 mm i.d.) at 25 °C. Finally, injection volume for both analyses was set at 10 μL.

Ergothioneine and methimazole (I.S) were separated using a 15-min gradient elution program, which consisted of water with 0.1% formic acid (Solvent A) and acetonitrile (Solvent B) at a steady flow rate of 0.2 mL min^−1^. The gradient started with 30% of solvent A, increased to 50% over 10 min of analysis, and, in 15 min, the percentage of solvent A ramped to initial conditions (30%). On the other hand, lovastatin and simvastatin (I.S) were separated using a 10-min isocratic elution program, which consisted of water with 0.1% formic acid (Solvent A, 40%) and acetonitrile (Solvent B, 60%).

For ergothioneine analysis, a 3D quadrupole ion trap LCQ FLEET (Thermo Scientific, USA) mass spectrometer was used, while for lovastatin–where a detector of higher resolution was required– an LTQ Orbitrap Velos mass spectrometer (Thermo Scientific, USA) was utilized. Tandem mass spectrometry MS/MS measurements were performed in a positive mode using an electrospray chemical ionization (ESI) source at mass scan width of 100–350 *m*/*z* for ergothioneine and 250–550 *m*/*z* for lovastatin, respectively. The mass tolerance window for mass identification of product ions was set at ±5 ppm. Source parameters are fully described in Table 5. All spectra were processed by Xcalibur software (Version 3.0, Thermo Scientific, USA).

**Figure 4:**
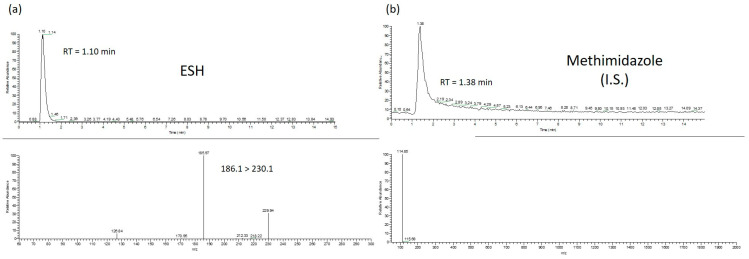
Representative chromatographs and mass spectra of Ergothioneine (**a**) and Methimidazole (internal standard) (**b**).

The identification and quantification of the two analytes under determination was based on the fragmentation of the precursor ions into the respective product ions using a single reaction monitoring (SRM) technique. More specifically, product fragments of ergothioneine with *m*/*z* = 186.1 (C8H15N3S) were observed (Figure 4a) at a retention time (RT) of 1.10 min. Based on the MS results and previous published data, these fragments are characteristic of the fragmentation of the precursor ion called ergothioneine [16]. An exception was the identification of methimazole for which only the precursor ion with *m*/*z* = 114.9, observed at a retention time of 1.38 min, was used for the analysis (Figure 4b).

**Figure 5:**
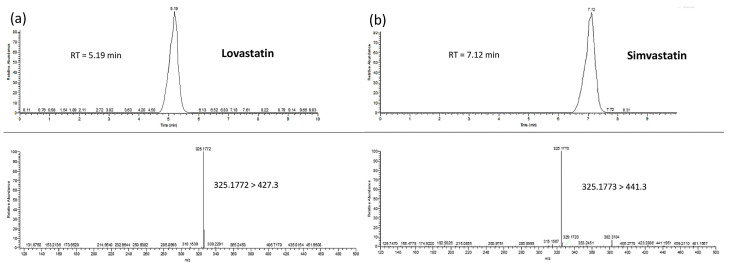
Representative chromatographs and mass spectra of Lovastatin (**a**) and Simvastatin (internal standard) (**b**).

Similarly, during the identification and quantification of lovastatin, a product ion of *m*/*z* = 325.1772 (C_17_H_21_ON_6_) was observed at a retention time of 5.19 min. Even though lovastatin and simvastatin were fragmented to the same product ion, a sufficient separation was achieved because the second compound was eluted at a different retention time of 7.12 min (Figure 5a). Taking into consideration the standard solutions analyzed and previous published results [52], this ion is characteristic of the fragmentation of the precursor ion of lovastatin and simvastatin (Figure 5b).

### 3.6. Liquid Chromatography–Mass Spectrometry Methods’ Validation

Liquid chromatography–mass spectrometry (LC-MS) methods’ validation was performed in terms of linearity, accuracy, intra-day (repeatability) and inter-day (reproducibility) precision, extraction recovery, and a matrix effect (ME). Validation runs were conducted on three consecutive days. The linearity was determined using fourteen ergothioneine standards with concentrations ranging from 0.05 to 45 μg mL^−1^ and ten lovastatin standards with concentrations ranging from 0.001 to 1 μg mL^−1^. The concentrations of ergothioneine, recorded in mushrooms, present higher variability and a wider concentration range than lovastatin. Thus, a calibration curve including a more extended range of concentrations were constructed in the case of ergothioneine (*n* = 14 instead of *n* = 10, in the case of lovastatin). Due to the wider selected concentration range, more concentration levels (*n* = 14) of the standard solutions were required to assure the linearity of ergothioneine’s calibration curve. Finally, in order to determine the detection (LoD) and quantification limits (LoQ) of the two developed methods, the guidelines of the Official Journal of the European Communities was adopted [53]. For that purpose, 0.01 μg mL^−1^ of ergothioneine and 0.001 μg mL^−1^ of the lovastatin standard were used, respectively.

For the estimation of intra-day precision and accuracy, three replicates (*n* = 3) of low, medium, and high concentrations of quality control (QC) samples were analyzed. More specifically for ergothioneine, QC samples of 5, 25, and 40 μg mL^−1^ were used, while, for lovastatin, QC samples of 0.005, 0.05, and 0.5 μg mL^−1^ were determined. The inter-day precision (or intermediate precision) was assessed by analysis of three batches of QC on three different days (*n* = 3 replicates per day, N = 3 different days for each concentration level). The precision was defined as the relative standard deviation (RSD%) and the accuracy was expressed as a relative error (RE%).

For the determination of the extraction recovery for ergothioneine and lovastatin, three different samples were analyzed: un-spiked mushroom samples (A), spiked mushroom samples with 10 μg mL^−1^ of ergothioneine, or 0.05 μg mL^−1^ of lovastatin (B) and standard solutions of these corresponding concentrations (C). The equation below (Equation (1)) was used to define the extraction recoveries.

(1) $$\mathrm{Recovery}\;=\;\frac{\left(B-A\right)}{C}\;\times \;100$$

The matrix effect (ME) estimation was conducted at low, medium, and high concentration levels by comparing the peak areas of each analyte spiked in mushroom samples with those of standard solutions at the same concentration. For that purpose, *A. bisporus* and *P. ostreatus* mushroom samples were pooled together while *P. citrinopileatus* mushroom samples, produced in three substrates, were examined separately. Peak areas of standard solutions were defined as A, whereas the peak areas of samples spiked with analyte were defined as B. The ratio below (Equation (2)) was used to evaluate the matrix effect.

(2) $$\mathrm{Matrix}\;\mathrm{Effect}\;=\;\frac{B}{A}\;\times \;100$$

### 3.7. Statistical Analysis

The statistical analysis of the results of the different analytical techniques was performed by one-way analysis of variance (ANOVA). In this study, the basic criterion for statistical significance, at a 95% confidence level, was *p-*value ≤ 0.05. For the calculation of the *p-*value, three measurements of the samples were included (*n* = 3).

## 4. Conclusions

Despite the fact that UV–Vis is a relatively inexpensive and rapid method, the outcome of the present work suggests that it should not be acknowledged as the most suitable technique for the identification and quantification of ergothioneine and lovastatin in mushrooms due to numerous restrictions imposed by the different co-existing mushroom constituents absorbing at the same wavelength or close wavelengths. Specifically, the phenolic profile and amino acids of mushrooms and cultivation substrates are considered to be the major factors affecting the accurate quantification of these compounds by spectrometric methods. The impact of these bioactive compounds on UV–Vis-determined ergothioneine and lovastatin content is more pronounced in the comparison of mushrooms from different cultivation substrates (GM and OL) rather than among mushroom species. Therefore, the possible application of this technique, could potentially provide misleading results regarding the selection of the most suitable substrate(s) for an ergothioneine-rich or lovastatin-rich mushroom production. Perhaps, the optimization of the implemented extraction methodologies or the replacement of the existing extraction techniques with more selective procedures resulting at higher product yields, could be an area of future investigation.

In contrast, the LC–MS methods implemented, which combined precision and higher sensitivity, showed significant differences in ergothioneine and lovastatin content (Table 3 and Table 4) in comparisons among species and substrates that were not observed during UV–Vis determination. Ergothioneine, although detected in all samples, was significantly higher in *P. citrinopileatus* mushrooms (822.1 (±20.6) mg kg^−1^ dry sample). In contrast, the lovastatin content in *A. bisporus* (1.39 (±0.014 mg kg^−1^ dry samples) was higher than in *Pleurotus* mushrooms. Nonetheless, lovastatin levels could be increased by using suitable/alternative cultivation substrates. In addition, *P. citrinopileatus* mushrooms produced on OL showed the highest levels of ergothioneine, (884.5 (±20.0) mg kg^−1^ dry sample), while fruitbodies from GM-based substrates contained the highest amounts of lovastatin (0.218 (±0.014) mg kg^−1^ dry sample).

Since non-conventional substrates seem to have an impact on the biosynthetic pathways and the final content of the examined compounds, the elucidation of the relationship between substrates’ content in other bioactive compounds (e.g., phenolics, amino acids) and ergothioneine or lovastatin yields would be an area of investigation. However, more mushroom strains/species and a wider range of substrates need to be studied to provide solid evidence confirming these assumptions.

## Figures and Tables

**Table 1:** Analytical figures of the merit of liquid chromatography–mass spectrometry (LC-MS) for ergothioneine (ESH) and lovastatin (LOV) determination.

Analytical Figures of Merit	ESH	LOV
Concentration range (μg mL^−1^)	0.05–45 (*n* = 14) ^1^	0.001–1 (*n* = 10) ^1^
Slope (a) (± standard error-sa)	0.0307 (±0.00023)	35.47 (±0.18)
Intercept (b) (± standard error-sb)	0.0012 (±0.0051)	0.090 (±0.065)
R^2^ (Correlation coefficient)	0.9993	0.9998
Limit of Detection-LoD (μg mL^−1^)	0.02	0.00039
Limit of Quantification LoQ (μg mL^−1^)	0.06	0.0012

^1^*n* = the number of the standard solutions of different concentrations tested.

**Table 2:** Precision, accuracy, and matrix effect of LC–MS method of ergothioneine and lovastatin.

Analyte		Quality Control Levels	
Ergothioneine	5.0 μg mL^−1^ (*n* = 3) ^2^	25.0 μg mL^−1^ (*n* = 3) ^2^	40 μg mL^−1^ (*n* = 3) ^2^
Intra–Day Precision (%RSD)	4.0	2.0	0.2
Inter–Day Precision (%RSD) N = 3 ^1^	7.5	1.9	2.5
Accuracy	102.95	100.95	99.67
Matrix Effect (%)	68.4	83.5	75.0
Lovastatin	0.005 μg mL^−1^ (*n* = 3) ^2^	0.05 μg mL^−1^ (*n* = 3) ^2^	0.5 μg mL^−1^ (*n* = 3) ^2^
Intra–Day Precision (%RSD)	13.6	4.91	4.04
Inter–Day Precision (%RSD) N = 3 ^1^	0.7	3.21	1.73
Accuracy	81.56	105.17	96.8
Matrix Effect (%)	42.08	15.9	8.3

^1^ N: The number of consecutive days for inter–day precision determination. ^2^ *n*: the number of QC replicates. RSD%: relative standard deviation.

**Table 3:** Ergothioneine–Lovastatin content in mushrooms of three species cultivated in conventional substrates by using ultraviolet-visible (UV–Vis) spectroscopy and LC–MS.

Method	Ergothioneine Content (mg kg^−1^ Dry Sample) ^a^		
	*A. bisporus*	*P. ostreatus*	*P. citrinopileatus*
UV–Vis	7100 (±300) ^c^	9200 (± 800) ^b^	8300 (±1100) ^b^
LC–MS	521.2 (±14.7) ^d^	607.3 (±11.2) ^c^	822.1 (±20.6) ^b^
**Method**	**Lovastatin Content (mg kg^−1^ Dry Sample) ^a^**		
UV–Vis	1050 (±80) ^b^	930 (±100) ^b^	840 (±250) ^b^
LC-MS	1.39 (±0.014) ^b^	1.11 (±0.042) ^c^	0.158 (±0.005) ^d^

^a^ Each value is expressed as mean ± standard error (*n* = 3), ^b–d^ Different letters within a row, indicate statistically significant differences at *p* < 0.05.

**Table 4:** Ergothioneine and lovastatin content of *P. citrinopileatus* mushrooms cultivated in three substrates, wheat straw (WS), grape marc (GM), and olive by-products (OL) by using UV–Vis and LC–MS.

Method	Ergothioneine Content (mg kg^−1^ Dry Sample) ^a^		
	WS	GM	OL
UV–Vis	8300 (±1100) ^b^	11800 (±1400) ^b^	6700 (±1100) ^b^
LC–MS	822.1 (±20.6) ^b^	637.2 (±24.5) ^c^	884.5 (±20.0) ^b^
**Method**	**Lovastatin Content (mg kg^−1^ Dry Sample) ^a^**		
UV–Vis	840 (±250) ^b^	860 (±180) ^b^	904 (±0.241) ^b^
LC-MS	0.158 (±0.005) ^c^	0.218 (±0.014) ^b^	0.161 (±0.009) ^c^

^a^ Each value is expressed as mean ± standard error (*n* = 3), ^b, c^ Different letters within a row, indicate statistically significant differences at *p* < 0.05

**Table 5:** Optimized values of electrospray chemical ionization (ESI) parameters for the examined compounds.

Source Parameters	Lovastatin-Simvastatin	Ergothioneine-Methimidazole
S-LENS RF Amplitude (V)	60	120
Sheath gas flow rate (arbitrary units, a.u)	8	7
Auxiliary gas flow rate (arbitrary units, a.u)	0	0
Sweep gas flow rate (arbitrary units, a.u)	0	0
Vaporizer temperature (°C)	320	300
Capillary temperature (°C)	220	200
Cone voltage (kV)	4	4.5
Isolation mass width	2	1.5
Collision energy (eV)	33 (lovastatin) 35 (simvastatin)	15 for ESH

## Data Availability

The data presented in this study are available on request from the corresponding author.

## Sample Availability

Not available.

## References

[1] Rathore H., Prasad S., Sharma S. (2017). Mushroom Nutraceuticals for Improved Nutrition and Better Human Health: A Review. Pharma Nutr..

[2] Gargano M.L., van Griensven L.J., Isikhuemhen O.S., Lindequist U., Venturella G., Wasser S.P., Zervakis G.I. (2017). Meidicinal mushrooms: Valuable biological resources of high exploitation potential. Plant Biosyst..

[3] Patel S., Goyal A. (2012). Recent Developments in Mushrooms as Anti-Cancer Therapeutics: A Review. 3 Biotech.

[4] Koutrotsios G., Patsou M., Mitsou E.K., Bekiaris G., Kotsou M., Tarantilis P., Pletsa V., Kyriakou A., Zervakis G.I. (2019). Valorization of olive by-products as substrates for the cultivation of *Ganoderma lucidum* and *Pleurotus ostreatus* mushrooms with enhanced functional and prebiotic properties. Catalysts.

[5] El Enshasy H.A., Hatti-Kaul R. (2013). Mushroom Immunomodulators: Unique Molecules with Unlimited Applications. Trends Biotechnol..

[6] Boulaka Α., Christodoulou P., Vlassopoulou M., Koutrotsios G., Bekiaris G., Zervakis G.I., Kyriacou A., Zervou M., Georgiadis P., Pletsa V. (2020). Genoprotective properties and metabolites of β-glucan-rich edible mushrooms following in vitro human faecal fermentation. Molecules.

[7] Muszyńska B., Grzywacz-Kisielewska A., Kała K., Gdula-Argasińska J. (2018). Anti-Inflammatory Properties of Edible Mushrooms: A Review. Food Chem..

[8] Kała K., Kryczyk-Poprawa A., Rzewińska A., Muszyńska B. (2020). Fruiting Bodies of Selected Edible Mushrooms as a Potential Source of Lovastatin. Eur. Food Res. Technol..

[9] Reis F.S., Barros L., Martins A., Ferreira I.C.F.R. (2012). Chemical Composition and Nutritional Value of the Most Widely Appreciated Cultivated Mushrooms: An Inter-Species Comparative Study. Food Chem. Toxicol..

[10] Atila F., Owaid M.N., Shariati M.A. (2017). The Nutritional and Medical Benefits of *Agaricus bisporus*: A Review. J. Microbiol. Biotechnol. Food Sci..

[11] FAOSTAT. http://www.fao.org/faostat/en/#data/TP/visualize.

[12] Royse D.J., Baars J., Tan Q. (2017). Edible and Medicinal Mushrooms: Technology and Applications.

[13] Kim M.Y., Lee S.J., Ahn J.K., Kim E.H., Kim M.J., Kim S.L., Moon H.I., Ro H.M., Kang E.Y., Seo S.H. (2009). Comparison of Free Amino Acid, Carbohydrates Concentrations in Korean Edible and Medicinal Mushrooms. Food Chem..

[14] Stampfli A.R., Blankenfeldt W., Seebeck F.P. (2020). Structural Basis of Ergothioneine Biosynthesis. Curr. Opin. Struct. Biol..

[15] Halliwell B., Cheah I.K., Tang R.M.Y. (2018). Ergothioneine–a Diet-Derived Antioxidant with Therapeutic Potential. FEBS Lett..

[16] Ey J., Schömig E., Taubert D. (2007). Dietary Sources and Antioxidant Effects of Ergothioneine. J. Agric. Food Chem..

[17] Lingappa K., Babu C.V., Siddalingeshwar K.G., Pramod T. (2004). Isolation, Screening and Rapid Confirmation of Lovastatin Producing Strains of Aspergillus Terreus. Indian J. Microbiol..

[18] Seenivasan A., Subhagar S., Aravindan R., Viruthagiri T. (2008). Microbial Production and Biomedical Applications of Lovastatin. Indian J. Pharm. Sci..

[19] Patel Y. (2012). Medicinal Properties of *Pleurotus* Species (Oyster Mushroom): A Review. World J. Fun. Plant. Biol..

[20] Goswami S., Vidyarthi A.S., Bhunia B., Mandal T. (2013). A Review on Lovastatin and Its Production. J. Biochem. Technol..

[21] Mulder K.C.L., Mulinari F., Franco O.L., Soares M.S.F., Magalhães B.S., Parachin N.S. (2015). Lovastatin Production: From Molecular Basis to Industrial Process Optimization. Biotechnol. Adv..

[22] Lisec B., Radež I., Žilnik L.F. (2012). Solvent Extraction of Lovastatin from a Fermentation Broth. Sep. Purif. Technol..

[23] Chen S.-Y., Ho K.-J., Hsieh Y.-J., Wang L.-T., Mau J.-L. (2012). Contents of Lovastatin, γ-Aminobutyric Acid and Ergothioneine in Mushroom Fruiting Bodies and Mycelia. LWT.

[24] Lo Y.-C., Lin S.-Y., Ulziijargal E., Chen S.-Y., Chien R.-C., Tzou Y.-J., Mau J.-L. (2012). Comparative Study of Contents of Several Bioactive Components in Fruiting Bodies and Mycelia of Culinary-Medicinal Mushrooms. Int. J. Med. Mushrooms.

[25] Lam Y.S., Okello E.J. (2015). Determination of Lovastatin, β-Glucan, Total Polyphenols, and Antioxidant Activity in Raw and Processed Oyster Culinary-Medicinal Mushroom, *Pleurotus ostreatus* (Higher Basidiomycetes). Int. J. Med. Mushrooms.

[26] Yuan H., Wang F., Tu J., Peng W., Li H. (2008). Determination of Lovastatin in Human Plasma by Ultra-Performance Liquid Chromatography–Electrospray Ionization Tandem Mass Spectrometry and Its Application in a Pharmacokinetic Study. J. Pharm. Biomed..

[27] Cheah I.K., Tang R.M.Y., Yew T.S.Z., Lim K.H.C., Halliwell B. (2017). Administration of Pure Ergothioneine to Healthy Human Subjects: Uptake, Metabolism, and Effects on Biomarkers of Oxidative Damage and Inflammation. Antioxid. Redox. Sign..

[28] González A.G., Herrador M.Á., Asuero A.G. (2010). Intra-Laboratory Assessment of Method Accuracy (Trueness and Precision) by Using Validation Standards. Talanta.

[29] Sapozhnikova Y., Brydwell W.C., Lobato A., Roming B. (2014). Effect of UV-B radiation levels on concentrations of phytosterols, ergothioneine and polyphenolic compounds in mushroom powders used as dietary supplements. J. Agric. Food Chem..

[30] Maftoun P., Johari H., Soltani M., Malik R., Othman N.Z., El Enshasy H.A. (2015). The Edible Mushroom *Pleurotus* Spp.: I. Biodiversity and Nutritional Values. Int. J. Biotechnol. Well. Indus..

[31] Alarcón J., Águila S. (2006). Lovastatin Production by Pleurotus Ostreatus: Effects of the C: N Ratio. Z. für Nat. C.

[32] Hajjaj H., Niederberger P., Duboc P. (2001). Lovastatin Biosynthesis by Aspergillus Terreus in a Chemically Defined Medium. Appl. Environ. Microbiol..

[33] Zhang Y., Chen Z., Wen Q., Xiong Z., Cao X., Zheng Z., Zhang Y., Huang Z. (2020). An Overview on the Biosynthesis and Metabolic Regulation of Monacolin K/Lovastatin. Food Funct..

[34] Koutrotsios G., Kalogeropoulos N., Kaliora A., Zervakis G.I. (2018). Toward an increased functionality in oyster (*Pleurotus*) mushrooms produced on grape marc or olive mill wastes serving as sources of bioactive compounds. J. Agric. Food Chem..

[35] Porterfield J.Z., Zlotnick A. (2010). A Simple and General Method for Determining the Protein and Nucleic Acid Content of Viruses by UV Absorbance. Virology.

[36] Hazra C., Samanta T., Mahalingam V. (2014). A Resonance Energy Transfer Approach for the Selective Detection of Aromatic Amino Acids. J. Mater. Chem. C.

[37] Mattila P., Salo-Väänänen P., Könkö K., Aro H., Jalava T. (2002). Basic Composition and Amino Acid Contents of Mushrooms Cultivated in Finland. J. Agric. Food Chem..

[38] Chirinang P., Intarapichet K.-O. (2009). Amino Acids and Antioxidant Properties of the Oyster Mushrooms, *Pleurotus ostreatus* and *Pleurotus sajor-caju*. Sci. Asia.

[39] Lafka T.-I., Sinanoglou V., Lazos E.S. (2007). On the Extraction and Antioxidant Activity of Phenolic Compounds from Winery Wastes. Food Chem..

[40] Leouifoudi I., Harnafi H., Zyad A. (2015). Olive Mill Waste Extracts: Polyphenols Content, Antioxidant, and Antimicrobial Activities. Adv. Phar. Sci..

[41] Rubilar M., Pinelo M., Shene C., Sineiro J., Nuñez M.J. (2007). Separation and HPLC-MS Identification of Phenolic Antioxidants from Agricultural Residues: Almond Hulls and Grape Pomace. J. Agric. Food Chem..

[42] Fotakis C., Kokkotou K., Zoumpoulakis P., Zervou M. (2013). NMR Metabolite Fingerprinting in Grape Derived Products: An Overview. Food. Res. Int..

[43] Xie X., Watanabe K., Wojcicki W.A., Wang C.C.C., Tang Y. (2006). Biosynthesis of Lovastatin Analogs with a Broadly Specific Acyltransferase. Chem. Biol..

[44] Li Y., Zhang F., Wang Z., Hu Z. (2004). Identification and Chemical Profiling of Monacolins in Red Yeast Rice Using High-Performance Liquid Chromatography with Photodiode Array Detector and Mass Spectrometry. J. Pharm. Biomed..

[45] Kontogianni V.G., Gerothanassis I.P. (2012). Phenolic Compounds and Antioxidant Activity of Olive Leaf Extracts. Nat. Prod. Res..

[46] Seenivasan A., Gummadi S.N., Panda T., Théodore T. (2015). Quantification of Lovastatin Produced by *Monascus purpureus*. Open Biotechnol. J..

[47] Lin S.-Y., Chen Y.-K., Yu H.-T., Barseghyan G.S., Asatiani M.D., Wasser S.P., Mau J.-L. (2013). Comparative Study of Contents of Several Bioactive Components in Fruiting Bodies and Mycelia of Culinary-Medicinal Mushrooms. Int. J. Med. Mushrooms.

[48] Weigand-Heller A.J., Kris-Etherton P.M., Beelman R.B. (2012). The Bioavailability of Ergothioneine from Mushrooms (*Agaricus bisporus*) and the Acute Effects on Antioxidant Capacity and Biomarkers of Inflammation. Prev. Med..

[49] Cohen N., Cohen J., Asatiani M.D., Varshney V.K., Yang Y.-C., Li Y.-H., Mau J.-H., Wasser S.P. (2014). Chemical composition and nutritional and medicinal value of fruit bodies and submerged cultured mycelia of culinary-medicinal higher basidiomycetes mushrooms. Int. J. Med. Mushrooms.

[50] Koutrotsios G., Mountzouris K.C., Chatzipavlidis I., Zervakis G.I. (2014). Bioconversion of Lignocellulosic Residues by *Agrocybe cylindracea* and *Pleurotus ostreatus* Mushroom Fungi–Assessment of Their Effect on the Final Product and Spent Substrate Properties. Food Chem..

[51] Ma L., Chen H., Zhu W., Wang Z. (2013). Effect of Different Drying Methods on Physicochemical Properties and Antioxidant Activities of Polysaccharides Extracted from Mushroom *Inonotus obliquus*. Food. Res. Int..

[52] Wujian J., Kuan-Wei P., Sihyung Y., Huijing S., Mario S., Zhuo W.M. (2015). A Simple Protein Precipitation-Based Simultaneous Quantification of Lovastatin and Its Active Metabolite Lovastatin Acid in Human Plasma by Ultra-Performance Liquid Chromatography-Tandem Mass Spectrometry Using Polarity Switching. J. Chromatogr. Sep. Tech..

[53] Union, P.O. of the E. CELEX1, 2002/657/EC: Commission Decision of 12 August 2002 implementing Council Directive 96/23/EC concerning the performance of analytical methods and the interpretation of results (Text with EEA relevance) (notified under document number C(2002) 3044). https://op.europa.eu/el/publication-detail/-/publication/ed928116-a955-4a84-b10a-cf7a82bad858/language-en.

